# Phosphate Transporter Profiles in Murine and Human Thymi Identify Thymocytes at Distinct Stages of Differentiation

**DOI:** 10.3389/fimmu.2020.01562

**Published:** 2020-07-22

**Authors:** Alice Machado, Marie Pouzolles, Sarah Gailhac, Vanessa Fritz, Marco Craveiro, Uriel López-Sánchez, Taisuke Kondo, Francesca Pala, Marita Bosticardo, Luigi D. Notarangelo, Vincent Petit, Naomi Taylor, Valérie S. Zimmermann

**Affiliations:** ^1^Pediatric Oncology Branch, Center for Cancer Research, National Cancer Institute, National Institutes of Health (NIH), Bethesda, MD, United States; ^2^Institut de Génétique Moléculaire de Montpellier, University of Montpellier, CNRS, Montpellier, France; ^3^Laboratory of Clinical Immunology and Microbiology, Division of Intramural Research, National Institute of Allergy and Infectious Diseases, NIH, Bethesda, MD, United States; ^4^Metafora-Biosystems, Paris, France

**Keywords:** thymus, phosphate transporters, glucose transporters, metabolism, human, mice, aging

## Abstract

Thymocyte differentiation is dependent on the availability and transport of metabolites in the thymus niche. As expression of metabolite transporters is a rate-limiting step in nutrient utilization, cell surface transporter levels generally reflect the cell's metabolic state. The GLUT1 glucose transporter is upregulated on actively dividing thymocytes, identifying thymocytes with an increased metabolism. However, it is not clear whether transporters of essential elements such as phosphate are modulated during thymocyte differentiation. While PiT1 and PiT2 are both phosphate transporters in the SLC20 family, we show here that they exhibit distinct expression profiles on both murine and human thymocytes. PiT2 expression distinguishes thymocytes with high metabolic activity, identifying immature murine double negative (CD4^−^CD8^−^) DN3b and DN4 thymocyte blasts as well as immature single positive (ISP) CD8 thymocytes. Notably, the absence of PiT2 expression on RAG2-deficient thymocytes, blocked at the DN3a stage, strongly suggests that high PiT2 expression is restricted to thymocytes having undergone a productive TCRβ rearrangement at the DN3a/DN3b transition. Similarly, in the human thymus, PiT2 was upregulated on early post-β selection CD4^+^ISP and TCRαβ^−^CD4^hi^DP thymocytes co-expressing the CD71 transferrin receptor, a marker of metabolic activity. In marked contrast, expression of the PiT1 phosphate importer was detected on mature CD3^+^ murine and human thymocytes. Notably, PiT1 expression on CD3^+^DN thymocytes was identified as a biomarker of an aging thymus, increasing from 8.4 ± 1.5% to 42.4 ± 9.4% by 1 year of age (*p* < 0.0001). We identified these cells as TCRγδ and, most significantly, NKT, representing 77 ± 9% of PiT1^+^DN thymocytes by 1 year of age (*p* < 0.001). Thus, metabolic activity and thymic aging are associated with distinct expression profiles of the PiT1 and PiT2 phosphate transporters.

## Introduction

The thymus is critical for the differentiation of T lymphocytes, promoting the generation of a pool of functionally competent T cells that provide protection against pathogens and tumors while maintaining self-tolerance. T cell differentiation in the thymus arises from progenitor cells that are derived from bone marrow hematopoietic stem cells (HSC) [reviewed in (1–5)]. Once progenitor cells enter into the thymus, the thymic environment generally results in their acquisition of a short-lived T cell precursor phenotype [as has been previously shown for common lymphocyte progenitors; (6, 7)]. Signals mediated through Notch1 (8), IL-7R (8), stem cell factor receptor (SCFR) (9) and CXCR4 (10) regulate the survival and proliferation of early T cell progenitors prior to the β-selection checkpoint. β-selection allows the differentiation of only those precursor T cells with productive, in-frame rearrangements of the TCRβ locus. In mice, β-selection occurs at a precise stage, within CD4^−^CD8^−^ double negative (DN) 3 (CD25^+^CD44^−^) thymocytes (9) whereas in humans, this step occurs in CD4^+^ intermediate single positive (ISP) cells as well as in double positive (DP) CD4^+^CD8α^+^CD8β^+^ thymocytes (11, 12). This TCR rearrangement results in a proliferative burst of murine as well as human thymocytes (13, 14), requiring an increased metabolism that is dependent on PI3K signaling downstream of Notch, IL-7, CXCR4 and the TCR (15–21).

Our understanding of the metabolic changes that regulate T cell differentiation and proliferation has generally focused on the roles of sugars, amino acids and fatty acids (22–24). However, it is clear that oxygen tension and pH balance, as well as minerals, vitamins, and electrolytes also participate to the metabolic crosstalk that occurs during T cell development. Indeed, the uptake of calcium (25–27) and iron (28) have long been known to be critical for T cell differentiation and more recently, potassium has been shown to regulate the effector function of T lymphocytes (29, 30).

The metabolic needs of proliferating cells are generally procured by an augmented entry of nutrients into the cells. Cell surface transporter expression is a rate-limiting step in nutrient entry and the induction of glucose, glutamine and other amino transporters are required for optimal T cell proliferation and effector function (31–36). In the context of differentiation within the thymus, we and others have demonstrated an upregulation of the GLUT1 glucose transporter on metabolically active murine as well as human thymocytes (14, 37, 38). Critically, the absence of thymic GLUT1 has been shown to result in a 60–70% loss of thymocytes (37). Interestingly though, the SLC1A5 glutamine transporter does not appear to be required for murine thymocyte differentiation (31–36, 39), possibly due to a redundancy with other glutamine transporters. However, the transferrin receptor, mediating iron delivery into differentiating thymocytes via transferrin, distinguishes metabolically active thymocytes and is required for thymocyte differentiation (14, 28, 40). Thus, several metabolite transporters play critical roles in the potential of an early thymocyte progenitor to differentiate to a mature T lymphocyte.

Notably though, the role of mineral transporters in T cell differentiation have not been extensively studied. Phosphorous is the sixth most abundant element in the human body and its anion phosphate is the most abundant, accounting for 1% of total body weight (41, 42). Humans take up approximately 16 mg/kg of phosphate per day from their diet via Na(+)-dependent SLC34 transporters that are expressed in the kidney and small intestine (42, 43). Transport into other cell types is regulated by the Na(+)-dependent PiT1/SLC20A1 and PiT2/SLC20A2 transporters (44, 45). Furthermore, more recently, an inorganic phosphate exporter, XPR1/SLC53A1 has also been identified (46). PiT1 and PiT2 share 60% sequence homology and in addition to a high affinity for *P*(i) (47, 48), they serve as retroviral receptors for the gibbon ape leukemia virus and koala endogenous retrovirus (49, 50) as well as the amphotropic murine leukemia virus (51, 52), respectively. PiT1 and PiT2 expression as well as their heterodimerization have been shown to be modulated by phosphate concentrations, at least in certain cell types (46, 53, 54).

While phosphate uptake by PiT1 and PiT2 are essential for bone homeostasis (42, 43), it is now clear that PiT1 and PiT2 play critical roles in multiple cell types. PiT1 is critical for survival as its deletion results in embryonic lethality at E12.5 due to severe anemia (55) while mutations in both PiT2 and XPR1 are associated with primary familial brain calcification (Fahr's disease) (56–59). Conditional deletions of PiT1 have revealed an important role for this transporter in cell proliferation and development (60–63), erythroid and B cell differentiation (64, 65) as well as inflammation and wound healing (66, 67). However, the thymic expression profiles of neither the PiT1 nor the PiT2 phosphate transporter are known. We hypothesized that the PiT1 and PiT2 transporters would display differential expression profiles, potentially allowing the identification of thymocytes with distinct maturation states. Here, we identify PiT2 as a marker of metabolically active thymocytes in both the murine and human thymus. In contrast, PiT1 distinguishes a CD3^+^DN subset of DN thymocytes that increases with age. We identify the majority of these cells as NKT thymocytes, thereby serving as a potential marker of age-related thymic atrophy. Thus, phosphate transporter expression identifies distinct thymocyte subsets, correlating murine and human thymocyte differentiation and identifying thymus populations that change as a function of age.

## Methods and Materials

### Mice and Cell Lines

C57Bl/6 mice were purchased from Charles River and maintained under specific pathogen-free conditions in the IGMM animal facility (Montpellier, France) or the NCI animal facility (Bethesda, MD). *Rag2*^−/−^ mice as well as Pmel-1 (B6.Cg-Thy1a/Cy Tg(TcraTcrb)8Rest/J) mice on a C57Bl/6 background were purchased from Jackson Laboratories. Unless indicated, mice were between 4 and 8 weeks of age. In indicated experiments, mice were 2 weeks, 8 weeks, or 1 year of age. All experiments were approved by the local animal facility institutional review boards. Animal care and experiments were performed in accordance with National Institutes of Health (NIH) and French national guidelines.

HAP1 cells, harboring a near-haploid genome, were derived from the chronic myelogenous leukemia (CML) cell line KBM-7 (68), and a gene edited HAP1 line with a deletion of SLC20A2/PiT2 was obtained from Horizon Discovery, as described (69). BxPC3, a pancreatic cancer cell line obtained from the ATCC, was used for shRNA-mediated knockdown of PiT1, as previously described (61).

### Thymocyte Preparation

Murine thymi were removed after sacrifice. Human thymi were removed during corrective cardiac surgery of pediatric patients aged 4 months−7 years at La Timone Hospital or from the pathology department of the Children's National Medical Center in Washington, DC following cardiothoracic surgery from children with congenital heart disease, as the thymic tissue is routinely removed and discarded to gain adequate exposure of the retrosternal operative field. Use of these thymus samples for this study was determined to be exempt from review by the NIH Institutional Review Board in accordance with the guidelines issued by the Office of Human Research Protections. All tissues were processed after isolation. Tissue was transferred to a sterile 10 mm^2^ tissue culture dish. Single cell thymocyte suspensions were generated by physical disruption of tissue and filtration through 70 μm nylon screens.

### Flow Cytometry

Murine thymocytes were stained with the following directly conjugated mAbs; CD3, CD25, CD8, CD71, c-Kit, CD44, CD11b, CD19, Ter119, Gr1, PD1, CD4, TCRγδ and NK1.1 (from Becton Dickinson, BioLegend or eBiosciences). Human thymocytes were stained with the following directly conjugated mAbs; CD8α, CD4, CD33, CD19, CD56, GlyA, TCRαβ, and CD71. Cells that were not thymocytes were eliminated with a dump including mAbs against CD19, Gr1, CD11b, and Ter119 for murine samples and CD19, CD33, ahd GlyA for human samples. Soluble ligands derived from the receptor binding domains (RBDs) of the HTLV, koala endogenous retrovirus (Ko-RBD) and mouse amphotropic-MLV (A-RBD) retrovirus were used to detect expression of their respective receptors; GLUT1, PiT1, and PiT2, as previously described (46, 70, 71) (Metafora biosystems). Stained cells were analyzed by flow cytometry (FACS-Canto II or LSR II-Fortessa, Becton Dickinson, San Jose, CA) and 1–2 × 10^e6^ events/sample were routinely acquired. The gating strategies for human and murine thymocytes are shown in Supplementary Figure 1. When indicated, molecules of equivalent soluble fluorochrome (MESF) were evaluated by Quantum MESF beads (Bang Laboratories, Fisher Indiana). Delta geometric mean fluorescence intensity (dGeo MFI) was calculated as the Geometric MFI of specific staining minus the Geometric MFI of the FMO. Data analyses were performed using Diva (BD Biosciences), and FlowJo Mac v.10.6.2 software (Tree Star).

### Statistical Analyses

Data were analyzed using GraphPad software version 8 (Graph Pad Prism, La Jolla, CA) and *p*-values were calculated using unpaired *t*-tests and one- or two-way ANOVA (Tukey's multiple comparison test), as indicated. *P*-values for comparisons of all conditions in the different figure panels are presented in the figure legends.

## Results

### Surface Expression of the GLUT1 and PiT2 Transporters Characterizes ISP Murine Thymocytes

GLUT1 has previously been shown to be expressed on metabolically active murine and human thymocytes, with cell surface levels exhibiting significant differences as compared to mRNA or even intracellular protein levels (14, 37). This is critical as it is the rapid translocation of solute carriers from intracellular stores to the cell surface that reflects the cell's response to extracellular stimuli; this has been extensively described for the insulin-mediated induction of GLUT1/GLUT4 to the cell surface within minutes of stimulation (72, 73). However, measurements of the cell surface expression of multipass transmembrane proteins such as SLC2A1/GLUT1 and the phosphate importers (PiT) have been hindered by a paucity of reliable antibodies, due to sequence conservation and poor immunogenicity of extracellular loops (74). Here, we utilized tagged receptor binding domain (RBD) fusion proteins from the HTLV (H2-RBD), Koala endogenous retrovirus (Ko-RBD) and mouse amphotropic MLV retrovirus (A-RBD) to specifically detect expression of GLUT1, PiT1, and PiT2, respectively, as previously shown (46, 69–71, 74–77). The specificity of H2-RBD binding to GLUT1 has previously been reported (74, 76) and the specificity of Ko-RBD and A-RBD to PiT1 and PiT2, respectively, were evaluated as a function of shRNA-mediated knockdown and CRISPR gene editing (Supplementary Figure 2) (69).

**Figure 1:**
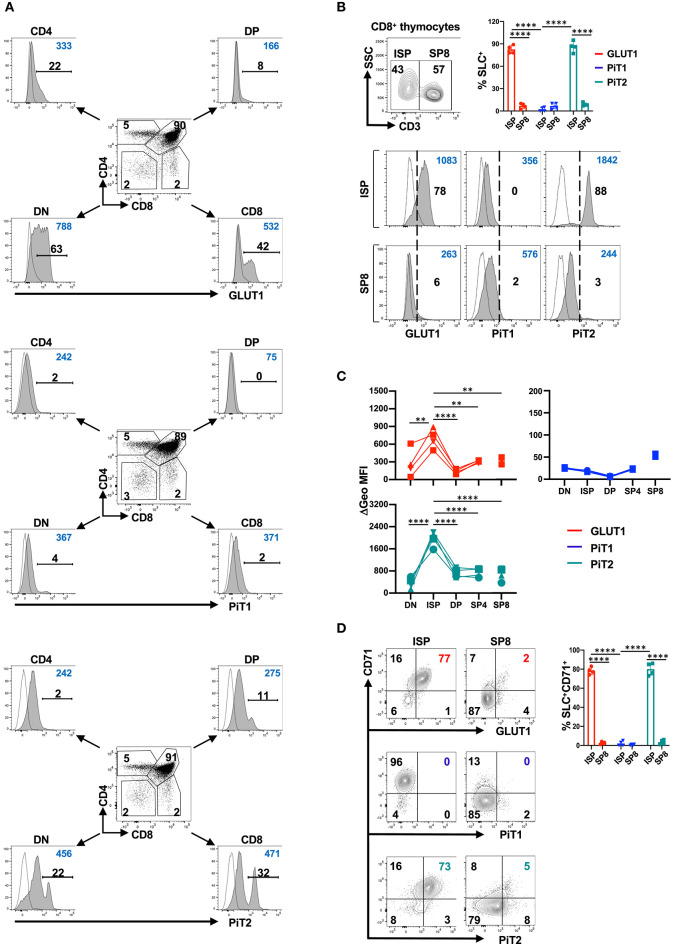
The PiT2 phosphate transporter is co-expressed with GLUT1 and CD71 transporters in the murine thymus. **(A)** Surface expression of the GLUT1 glucose transporter as well as PiT1 and PiT2 phosphate transporters were evaluated on murine thymocytes as a function of their CD4/CD8 profiles. Mean fluorescence intensity (MFI) on double negative (DN), double positive (DP), and single positive (SP) CD4 and CD8 thymocytes is shown for each transporter. Specific staining is shown in gray histograms and non-specific FMO staining is shown as dotted line histograms. The percentages of positively stained cells (black) and geometric MFIs (blue) in each histogram are indicated. Data are representative of one of eight individual thymi. **(B)** The distribution of CD3^−^CD8^+^ intermediate single positive (ISP) thymocytes and mature CD3^+^ SP8 thymocytes within the CD8^+^ thymocyte gate was evaluated as a function of CD3 staining and a representative dot plot is presented (top). GLUT1, PiT1 and PiT2 staining within the ISP and SP8 gates are presented relative to non-specific staining and percent staining (black) as well as geometric MFIs (blue) are indicated in each histogram (bottom). Data are representative of one of eight individual thymi and quantification of transporter (solute carrier, SLC) expression in ISP8 and SP8 thymocytes is shown. **(C)** Delta geometric MFIs of GLUT1 (red), PiT1 (blue) and PiT2 (green) staining in DN, ISP, DP, SP4, and SP8 subsets are presented for four individual thymi. **(D)** The phenotype of ISP and SP8 thymocytes was evaluated as a function of GLUT1, PiT1, and PiT2 transporters and the CD71 transferrin receptor and the percentages of cells in the different quadrants are indicated (left panel). Quantification of the percentages of solute carrier (SLC) ^+^ cells co-expressing CD71 in ISP and SP8 thymocytes are presented (*n* = 4, right panel). **p < 0.01; ****p < 0.0001.

Within the murine thymus, analyses of immature DN, DP, and single positive CD4 and CD8 thymocytes revealed the presence of subpopulations of GLUT1^+^ and PiT2^+^ cells within the DN and CD8^+^ thymocyte gates (Figure 1A). In contrast, distinct subsets of PiT1^+^ cells were not easily detected (Figure 1A). To evaluate the identity of the GLUT1^+^ and PiT2^+^ CD8 thymocyte subset, we assessed whether these transporters were expressed in the immature CD3^−^ population or the mature CD3^+^ population. As shown in Figure 1B, the vast majority of GLUT1^+^ as well as PiT2^+^ cells were immature ISP thymocytes while PiT1 expression on CD8 thymocytes was not detected (*p* < 0.0001). Moreover, the geometric mean of GLUT1 and PiT2 expression decreased significantly between ISP8 and SP8 thymocytes, from 699 ± 167 to 330 ± 24 and 1970 ± 277 to 680 ± 237, respectively (*p* = 0.003 and *p* < 0.0001, respectively, Figures 1B,C). Expression of the CD71 transferrin receptor is often a marker of a cell's metabolic activity and it has been shown to be co-expressed with GLUT1 in the human thymus (14, 28). We therefore evaluated transporter profiles as a function of CD71 expression in ISP8 as compared to SP8 thymocytes. Notably, CD71 expression was largely confined to the ISP subset and was co-expressed by both GLUT1^+^ and PiT2^+^ thymocytes (78 ± 4% vs. 3 ± 1%, *p* < 0.0001 and 80 ± 6% vs. 4 ± 2%, *p* < 0.0001, respectively; Figure 1D). In contrast, PiT1 was not detected on the CD71^+^ISP subset (2 ± 2%). Together, these results demonstrate a strong association of GLUT1 and PiT2 on metabolically active murine ISP thymocytes (Figure 1D).

While GLUT1^+^ and PiT2^+^ subsets were not clearly discerned in the CD4 gate, we specifically evaluated regulatory T cell (Treg) thymocytes in the Foxp3^+^CD4^+^ subset. Approximately 50% of Foxp3^+^ thymocytes were CD25^−^ as compared to CD25^+^, representing immature and mature Treg subsets, respectively (78). Interestingly, while similar percentages of CD25^−^Foxp3^+^ and CD25^+^Foxp3^+^ thymocytes expressed GLUT1 (25 ± 8% of CD25^−^ and 29 ± 7% of CD25^+^, respectively), PiT2 expression was significantly higher in CD25^+^ Treg (20 ± 7% vs. 39 ± 10%, *p* < 0.01, Supplementary Figure 3). The significance of PiT2 expression on CD25^+^Foxp3^+^ thymocytes remains to be determined.

### PiT2 Expression Distinguishes Metabolically Active TN Thymocyte Subsets

**Figure 2:**
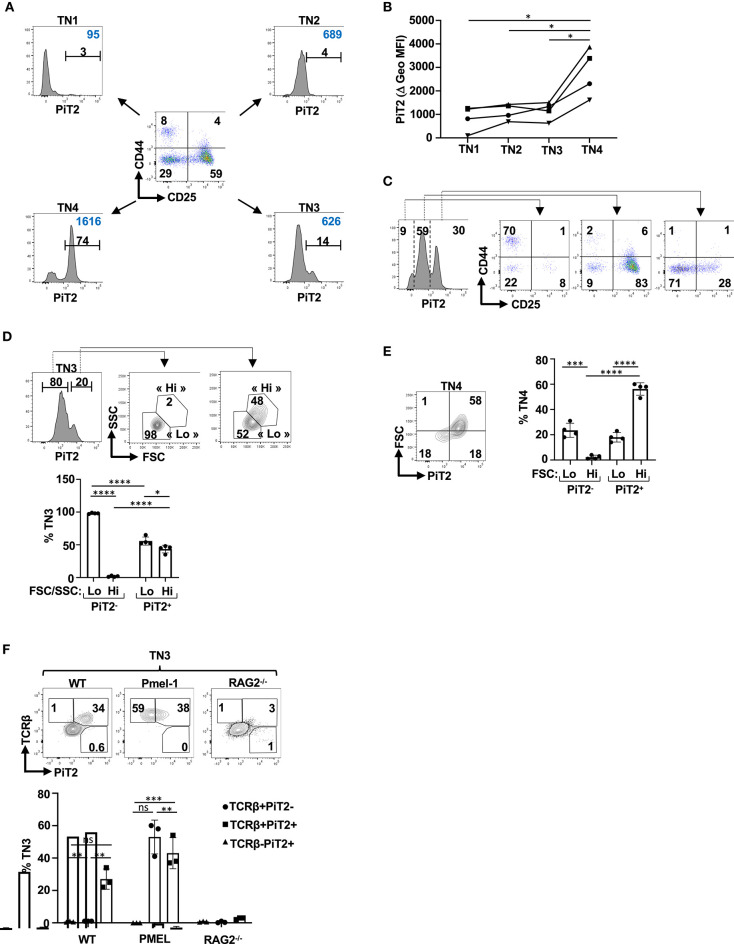
β selection of TN thymocytes results in the induction of the PiT2 phosphate transporter. **(A)** Profiles of TN thymocytes were evaluated as a function of their CD44/CD25 profiles and a representative dot plot showing the TN1 (CD44^+^CD25^−^), TN2 (CD44^+^CD25^+^), TN3 (CD44^−^CD25^+^), and TN4 (CD44^−^CD25^−^) subsets is presented. Histograms of PiT2 expression in each TN subset is shown. The percentages of positively staining cells (black) and geometric MFI (blue) are presented. **(B)** PiT2 staining in TN1, TN2, TN3 and TN4 thymocytes from four individual thymi are presented as a function of their geometric MFI. **(C)** CD44/CD25 profiles of TN thymocytes distinguished as a function of their PiT2 levels are presented. **(D)** TN3 thymocytes were distinguished on the basis of PiT2 expression and FSC/SSC profiles of PiT2-negative and PiT2^+^ cells are presented. The percentages of FSC/SSC-lo and FSC/SSC-hi subsets, used to distinguish TN3a and TN3b thymocytes, are presented (top). Quantification of the percentages of cells within the FSC/SSC-lo and FSC/SSC-hi gates are presented for PiT2-negative and PiT2-positive TN3 thymocytes (*n* = 4, bottom). **(E)** Expression of PiT2 in TN4 thymocytes was evaluated as a function of FSC and a representative plot is presented (right). Quantification of the percentages of FSC-lo and FSC-hi TN4 thymocytes are shown as a function of PiT2 expression (*n* = 4, right). **(F)** Representative histograms showing PiT2 staining on TN3 thymocytes from WT, Pmel-1 TCR transgenic and *Rag2*^−/−^ thymocytes are shown as a function of TCRb expression (top panels). Quantification of the percentages of PiT2^+^ cells in the TCRb^−^ and TCRb^+^ TN3 subsets are shown for WT, Pmel-1, and *Rag2*^−/−^ mice. Statistical differences were evaluated by a 2-tailed unpaired *t*-test. *p < 0.05; **p < 0.01; ***p < 0.001; ****p < 0.0001.

The differentiation of CD4^−^CD8^−^CD3^−^ (TN) thymocytes has been historically divided into four subsets on the basis of CD44 and CD25 expression, with TN1, TN2, TN3 and TN4 subsets defined as CD44^+^CD25^−^, CD44^+^CD25^+^, CD44^−^CD25^+^ and CD44^−^CD25^−^, respectively (1, 2, 4). Given the heterogeneity of PiT2 expression in the DN thymocyte subsets (Figure 1A), we specifically evaluated PiT2 levels in each of the TN subsets. As shown in Figure 2A, PiT2 levels were heterogeneous even within specific TN subsets but high levels were detected mainly within TN3 and TN4 subsets, evaluated as a function of percent positively stained thymocytes and the MFI of staining. Indeed, MFI increased significantly between TN3 and TN4 thymocytes (*p* < 0.05, Figure 2B); the vast majority of TN1 thymocytes were PiT2-negative, whereas TN3 and TN4 thymocytes were detected in both the PiT2-intermediate and PiT2-high gates (Figure 2C).

β-selection, the first checkpoint in thymocyte development, occurs at the TN3 stage. Only TN3 thymocytes expressing a functional pre-TCR proliferate and progress to the DP stage of thymocyte differentiation (13, 79). Those TN3 and TN4 cells that have undergone a productive TCRβ gene rearrangement have been historically distinguished from the majority of TN3/TN4 cells with random TCRβ gene rearrangements by their size, monitored as a function of forward and side scatters (FSC/SSC) (13). Furthermore, TN3/TN4 thymocytes are activated through PI3K/Akt signaling (15, 17, 20, 21) resulting in the induction of metabolic transporters such as the GLUT1 glucose transporter (14) as well as chemokine receptors such as CXCR4 (14, 21). We therefore evaluated whether expression of PiT2 in TN thymocytes allows a discrimination of β-selection. Notably, 98 ± 1% PiT2-negative TN3 thymocytes were characterized as FSC/SSC-lo and <2% as FSC/SSC-hi (*p* < 0.0001, Figure 2D). Thus, PiT2 negative cells appear to represent a TN3a profile (FSC-lo). Interestingly though, within the PiT2^+^ gate, TN3 thymocytes exhibited both FSC/SSC-lo and FSC/SSC-hi profiles, with 56 ± 6% and 43 ± 4%, respectively. Furthermore, within the TN4 gate, PiT2 distinguished FSC-lo and FSC-hi cells. Within the PiT2-negative TN4 subset, only 2 ± 1% were FSC-hi while similarly to the TN3 population, PiT2^+^ TN4 thymocytes were both FSC-lo and FSC-hi (18 ± 4% and 56 ± 5%, respectively, Figure 2E). Thus, even though the percentages of PiT2^+^ cells that are FSC-hi are significantly higher than those that are FSC-lo (*p* < 0.0001), the presence of an FSC-lo subset suggests PiT2 expression might allow for a more rigorous identification of TN3 and TN4 thymocyte subsets that have undergone TCRβ gene rearrangement. Specifically, PiT2 expression may be a marker of those TN thymocytes that have responded to TCR/CXCR4 signaling.

To directly address this point, we evaluated PiT2 profiles within the TN subset of WT, RAG2-deficient and Pmel-1 thymi. In the absence of RAG2, thymocytes are blocked at the TN3 stage of differentiation as they are not able to rearrange a functional pre-TCR (80, 81) while Pmel-1 thymocytes, harboring a transgenic TCR against the gp100 melanoma antigen, do not need to undergo TCR rearrangement for their selection (82) (Supplementary Figure 4). Importantly, while a delineated peak of PiT2^+^TCRb^+^ TN3 thymocytes was detected in WT thymi (27 ± 6%), this was not the case in RAG2-deficient thymi (3 ± 1%, *p* < 0.0001; Figure 2F). Furthermore, in the Pmel-1 thymus where all TN3 thymocytes were TCRb^+^, a similar percentage of thymocytes as in WT mice expressed the PiT2 phosphate importer (Figure 2F). Conversely, PiT2 expression was not detected on TCRb^−^ TN3 thymocytes in either WT or RAG2^−/−^ mice (Figure 2F). Collectively, these data reveal the importance of the PiT2 phosphate transporter in identifying TCRb^+^ TN3 cells that have undergone a productive TCRβ gene rearrangement.

### PiT1 and PiT2 Expression Profiles Characterize Distinct Subsets of Human Thymocytes

**Figure 3:**
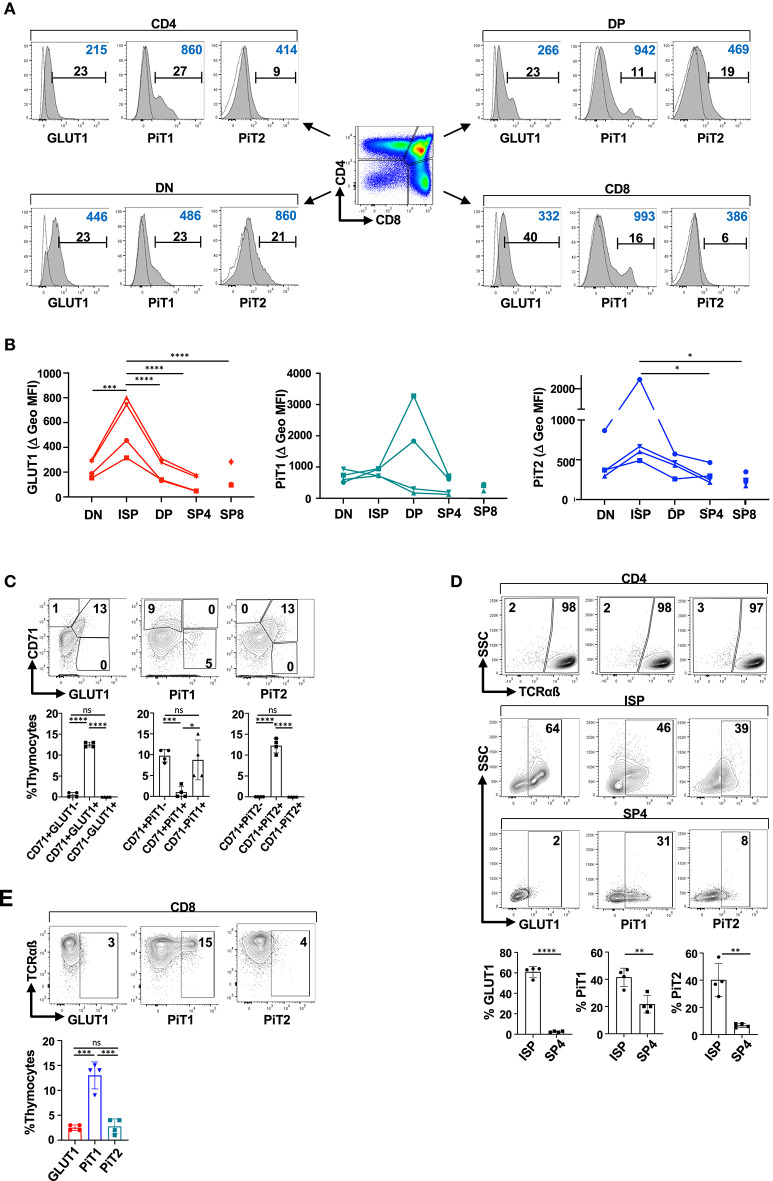
PiT2 expression identifies metabolically active TCRαβ^−^ thymocytes in the human thymus. **(A)** Surface expression of GLUT1, PiT1, and PiT2 transporters was evaluated on freshly isolated human thymocytes and representative histograms are shown as a function of their CD4/CD8 profiles. Specific staining is shown in gray histograms and non-specific FMO staining is shown as dotted line histograms. The percentages of positively stained cells (black) and geometric MFIs (blue) in each histogram are indicated. **(B)** Delta geometric MFIs of GLUT1, PiT1, and PiT2 staining in DN, DP, ISP, SP4, and SP8 subsets are presented from four analyses. **(C)** Expression of GLUT1, PiT1, and PiT2 were evaluated as a function of the CD71 transferrin receptor and representative dot plots are presented (top). The relative percentages of thymocytes co-expressing the indicated transporter are quantified and means ±SD are shown (bottom). **(D)** Immature CD4^+^ ISPs and mature SP4 thymocytes were differentiated on the basis of TCRαβ expression (top). Expression of GLUT1, PiT1, and PiT2 within TCRαβ^−^ ISP and TCRαβ^+^ SP4 thymocytes are presented (middle plots). Quantification of transporters within ISP and SP4 subsets are shown (bottom). **(E)** GLUT1, PiT1, and PiT2 expression were evaluated in SP8 thymocytes as a function of TCRαβ expression. Representative dot plots are presented. Statistical differences were evaluated by a 2-tailed unpaired *t*-test. *p < 0.05; **p < 0.01; ***p < 0.001; ****p < 0.0001.

T cell differentiation in both mouse and humans occurs in the thymus, proceeding through discrete developmental stages [reviewed in (83)]. While many of the same markers have been used to characterize murine and human differentiation, including CD4 and CD8, some differ and the ISP stage in humans is characterized as CD3^−^CD4^+^CD8^−^ (as compared to CD3^−^CD4^−^CD8^+^ in mice) (83). It was therefore important to determine whether the profiles of the PiT1 and PiT2 phosphate transporters in human thymocytes were similar to that detected in mice. Interestingly, while PiT1 was detected in only very low levels in the murine thymus, equivalent percentages of human thymocytes expressed surface GLUT1, PiT1, and PiT2 transporters (11 ± 1%, 10 ± 2%, and 10 ± 2%, respectively; Supplementary Figure 5). However, it is important to note that the profiles of expression on DN, DP, SP4, and SP8 thymocyte subsets was distinct (Figure 3A).

GLUT1 and PiT2 profiles were similar in the murine thymus, reflecting metabolically active thymocytes that had undergone a productive TCRβ gene rearrangement (Figure 2). Indeed, the MFI of GLUT1 and PiT2 staining was significantly higher on ISPs than on other subsets, decreasing from 579 ± 232 to 109 ± 71 and 1103 ± 828 to 306 ± 111 between ISP4 and SP4, respectively (*p* > 0.0001 and *p* > 0.05, Figure 3B). Thus, we assessed whether GLUT1 and PiT2 expression in the human thymus was associated with expression of the CD71 transferrin receptor, as in the murine thymus (Figure 1). Indeed, as shown in Figure 3C, almost all CD71^+^ thymocytes were GLUT1^+^ and PiT2^+^, representing 13 ± 1% and 12 ± 2% of total thymocytes, respectively (only 0.5 ± 0.6% and 0% of all CD71^+^ thymocytes were GLUT1^−^ or PiT2^−^, respectively). Interestingly though, only 1 ± 2% of PiT1^+^ thymocytes co-expressed CD71 while 9 ± 5% of PiT1^+^ thymocytes were CD71-negative (Figure 3C). Furthermore, within the CD4SP population, GLUT1^+^ and PiT2^+^ cells were almost exclusively within the ISP thymocyte subset (61 ± 5% and 40 ± 7%) as compared to the mature SP4 subset (3 ± 1% and 7 ± 2%, respectively; *p* < 0.0001 and *p* < 0.01, Figure 3D). Thus, similarly to the murine thymus, both GLUT1 and PiT2 expression are detected on metabolically active thymocyte subsets that have been signaled following TCRβ gene rearrangement.

In contrast with GLUT1 and PiT2, PiT1 expression was detected on mature SP4 as well as SP8 thymocytes. Within the SP8 thymocyte subset, 13 ± 3% of TCR^Hi^SP8 thymocytes were characterized by high surface PiT1 expression, and conversely, <3% of these cells expressed GLUT1 or PiT2 (3 ± 1% and 3 ± 2%, respectively, Figure 3E). These data point to important similarities in metabolite transporter expression in human thymocytes, especially as concerns those transporters that function as biomarkers for metabolic activity. However, there are also differences in transporter profiles between the species, especially as regards PiT1 levels, that remain to be evaluated.

### NKT Thymocytes Expressing the PiT1 Transporter Are a Biomarker of Thymic Aging

**Figure 4:**
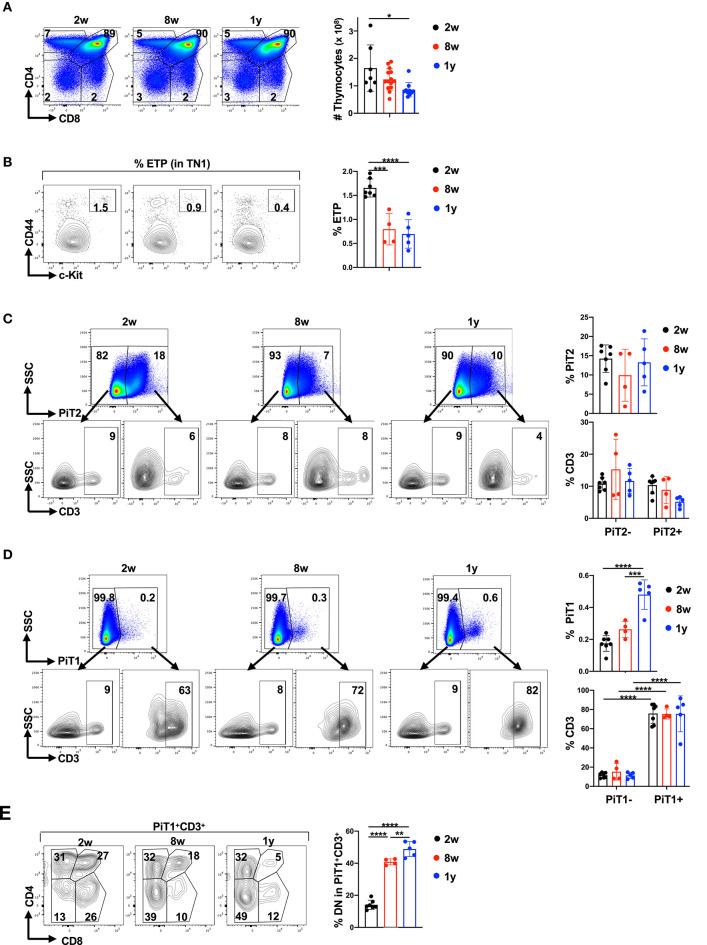
PiT1 but not PiT2 expression increases in CD3^+^DN thymocytes as a function of age. **(A)** Total thymocyte numbers were evaluated in mice at 2 weeks (2w), 8 weeks (8w), and 1 year (1y) of age. Representative CD4/CD8 profiles (top left) and quantification of total thymocytes (top right) are presented (*n* = 7–14 per age group). **(B)** The percentages of early thymic progenitors (ETP) within the TN1 gate were evaluated by CD44 and c-Kit expression and representative dot plots (left) as well as quantifications (right) are presented. **(C)** The percentages of thymocytes expressing PiT2 were evaluated as a function of age and representative plots are presented (top). The percentages of CD3^+^ thymocytes within the PiT2^−^ and PiT2^+^ subsets are presented (bottom) and quantifications are shown (*n* = 4–7 per age group, left). **(D)** The percentages of thymocytes expressing PiT1 were evaluated as a function of age (top) and CD3^−^ and CD3^+^ subsets representative plots (bottom) as well as quantifications (right) are presented. **(E)** The phenotype of PiT1^+^CD3^+^ thymocytes was evaluated as a function of their CD4/CD8 profiles (left) and the percentages of DN thymocytes within the PiT1^+^CD3^+^ subset is quantified for the different age groups (right). Statistical differences were evaluated by a one-way ANOVA test. *p < 0.05; **p < 0.01; ***p < 0.001; ****p < 0.0001.

Thymic function declines with age, due to changes in the thymic environment itself as well as to a decrease in the entry of BM-derived precursors that support thymopoiesis (84, 85). As such, we evaluated the impact of age on thymocyte subsets and more specifically, on the expression of phosphate transporters on these subsets. As previously shown (86–88), thymocyte numbers (Figure 4A) as well as c-Kit^+^ early thymic precursors decreased with age, diminishing from 1.7 ± 0.2% to 0.7 ± 0.3% between 2 weeks and 1 year of age (*p* < 0.0001; Figure 4B).

Given the decreased thymopoiesis in aging mice, we hypothesized that phosphate transporter profiles in the thymus would change with age. While we did not detect significant changes in the overall percentages of PiT2^+^ thymocytes as a function of age (Figure 4C), the percentages of PiT2 expression in the mature CD3^+^ subset was decreased by 1 year of age (from 10 ± 3% to 5 ± 2%, *p* < 0.01, Figure 4C). However, in marked contrast, the percentages of PiT1 expression increased significantly with age, from 0.1 ± 0.03% to 0.5 ± 0.1% (*p* < 0.0001; Figure 4D). Moreover, at all ages, the expression of PiT1 was significantly higher in CD3^+^ subset as compared to the CD3^−^ subset, with a difference of 75 ± 19 to 12 ± 4 at 1 year of age (*p* < 0.0001, Figure 4D). Within the CD3^+^PiT1^+^ thymocyte subset we evaluated the percentages of DN, DP and SP thymocytes and found that the percentages of PiT1^+^CD3^+^ thymocytes that were DN increased significantly over time, from 14.1 ± 2.8% to 48.9 ± 1.8%, respectively (*p* < 0.0001; Figure 4E). Thus, increased PiT1 expression on CD3^+^ thymocytes is a marker of thymic aging.

**Figure 5:**
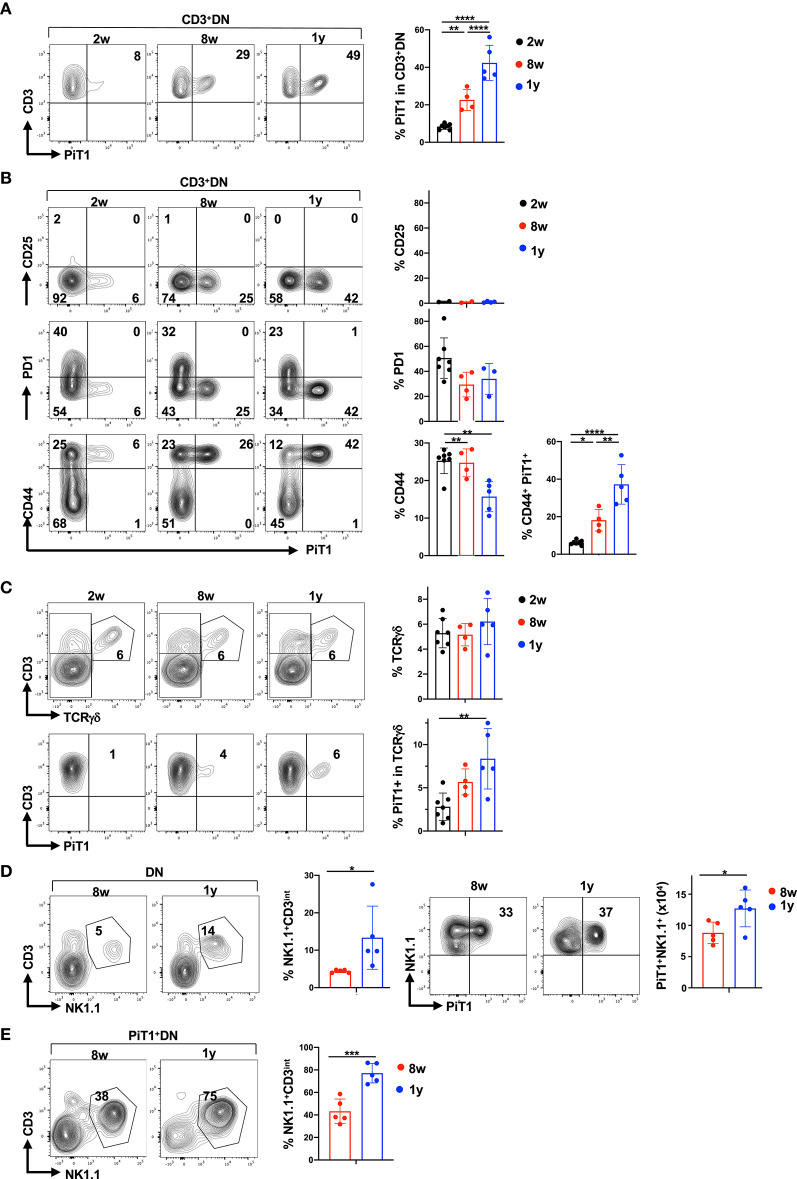
Thymic aging is associated with a significant increase in PiT1^+^ NKT thymocytes. **(A)** PiT1 expression in CD3^+^DN thymocytes is shown as a function of age (left) and quantifications in the different age groups are presented (right). **(B)** PiT1^+^CD3^+^DN thymocytes were evaluated as a function of CD25, CD71, PD1, and CD44 expression and representative profiles are presented (left). Quantifications of each staining profile in the different age groups are shown (right). **(C)** TCRγδ thymocytes were evaluated in the DN population by CD3/TCRγδ staining (top) and the percentages of PiT1 expressing TCRγδ T cells are presented (bottom). Quantifications are presented at the right. **(D)** NKT and NK thymocytes were evaluated in the DN population as a function of their CD3/NK1.1 staining profiles and representative plots at 8 weeks and 1 year are shown (left). Expression of PiT1 in NK1.1^+^ thymocytes are presented (left) and quantifications in 8 weeks and 1 year old mice are shown (*n* = 5). **(E)** The percentages of NKT thymocytes within the PiT1^+^DN population are shown as a function of CD3/NK1.1 staining (left) and quantifications for 8 weeks and 1 year mice are shown (*n* = 5, right). Statistical differences were evaluated by a one-way ANOVA test. *p < 0.05; **p < 0.01; ***p < 0.001; ****p < 0.0001.

We next specifically monitored PiT1 expression in the CD3^+^DN thymocyte gate and found that with age, the percentages of PiT1^+^ thymocytes increased massively—from 8.4 ± 1.5% at 2 weeks to 22.6 ± 5.6% at 8 weeks and further increasing to 42.4 ± 9.4% at 1 year (*p* < 0.0001; Figure 5A). To further characterize these PiT1^+^CD3^+^DN thymocytes, we monitored expression of the CD25, CD71, PD1, and CD44 markers. Notably, PiT1^+^CD3^+^DN thymocytes were CD25-negative and were not likely to be metabolically active as they did not express the CD71 transferrin receptor (Figure 5B and Supplementary Figure 6). Indeed, in accord with a reduced metabolism in the aging thymus, the percentage of PiT1^−^CD3^+^DN expressed CD71 decreased from 26 ± 11% to 9 ± 9% between 2 weeks and 1 year of age (*p* < 0.05, Supplementary Figure 6). Furthermore, these cells are unlikely to represent autoreactive thymocytes that have not undergone clonal deletion because this subset of CD3^+^DN thymocytes has been shown to be PD1^+^ (89) and the PiT1^+^CD3^+^DN subset did not express detectable levels of PD1 (Figure 5B). Importantly, all thymocytes in the PiT1^+^CD3^+^DN subset co-expressed CD44, increasing from 6 ± 1% to 38 ± 11% between 2 weeks and 1 year of age (*p* < 0.0001, Figure 5B).

CD44 expression is a marker in CD3^+^DN thymocytes of both γδ and NKT thymocytes (90–94). The percentages of thymocytes harboring a γδ TCR was not altered with age (5.5 ± 1.4%) but the percentages of PiT1^+^ TCR γδ thymocytes did increase, from 2.8 ± 1.6% to 8.4 ± 3.5% between 2 weeks and 1 year of age (*p* = 0.004; Figure 5C). Importantly though, PiT1 expression was detected on <15% of all γδ thymocytes. We therefore evaluated the evolution of NKT cells and found that they were significantly augmented in 1 year old mice, increasing from 4 ± 0.4% to 13 ± 8% (*p* < 0.05, Figure 5D). Moreover, despite the overall decrease in thymocyte numbers (Figure 4A), the number of PiT1^+^NKT increased from 3 × 10^5^ to 8 × 10^5^ (*p* < 0.05, Figure 5D) and NKT cells did not express either GLUT1 or PiT2 (Supplementary Figure 7A). We therefore evaluated whether NKT thymocytes accounted for a significant percentage of PiT1^+^DN thymocytes—while they accounted for 43 ± 11% of this subset at 8 weeks of age, the percentage increased to 77 ± 9% by 1 year of age (*p* < 0.001, Figure 5E). Thus, while markers of autoreactivity and metabolic activity decreased with age, PiT1 expression on γδ thymocytes and more notably on NKT cells appears to serve as phenotypic biomarkers of an aging thymus.

## Discussion

Metabolite transporters of the SLC superfamily comprise more than 400 genes, regulating the uptake of nutrients, vitamins, neurotransmitters, elements and ions [reviewed in (95)]. As such, they are likely to be critical components of all cell fate decisions, governing survival, proliferation, differentiation and function. While the impact of SLCs that transport nutrients such as glucose and amino acids have been extensively evaluated in T cell differentiation and function (35–37, 39, 96, 97), studies of anion-transporting SLCs have been more limited. Moreover, it is critical to evaluate cell surface expression of metabolite transporters as their induction is often regulated by translocation from intracellular compartments rather than by increased transcription and/or translation (98–100). Here, we show that PiT1/SLC20A1 and PiT2/SLC20A2, SLCs that have been characterized as ubiquitous “housekeeping” phosphate importers, are only expressed at the cell surface of a small percentage of thymocytes. Moreover, the two importers exhibit distinct cell surface expression profiles. In contrast with PiT1, PiT2 was detected on thymocytes with high metabolic activity, concomitant with expression of the GLUT1 and CD71 transporters as well as high FSC/SSC profiles. This profile, in both the murine and human thymus, identified immature thymocytes that had undergone a productive TCRβ rearrangement. Indeed, in the absence of RAG2, PiT2 expression was not upregulated, and its induction in TCRβ^+^ DN thymocyes shows that it serves as a biomarker of the DN3b/DN4 switch. Expression of PiT1, on the other hand, increased with age, exhibiting a significant increase on CD3^+^ DN thymocytes. We identified these cells to be PiT1^+^CD3^+^NK1.1^+^, revealing the association of PiT1^+^NKT thymocytes with thymic aging.

While we did not evaluate the impact of dynamic changes in PiT1 and PiT2 levels on phosphate uptake, it is important to note that these importers can regulate cell function and differentiation in a phosphate uptake-independent manner. Specifically, *PiT1*-null MEFs do not exhibit altered uptake of phosphate (60) and conditional deletion of PiT1, while significantly affecting erythroid and B cell differentiation in mice, does not decrease phosphate uptake in these cells (65). Moreover, while decreased PiT1 expression in tumor cells results in attenuated proliferation and tumor growth, these effects are independent of phosphate transport activity (61). That being said, depletion of extracellular phosphate is associated with an induction of both PiT1 and PiT2 on transformed cells (57) and it is therefore interesting to speculate that intrathymic phosphate levels decrease upon thymic involution, as a function of age. In that regard, intrathymic phosphate levels may themselves impact thymic involution—deletion of the *Klotho* gene, mediating the role of FGF-23 in the control of phosphate (101), has been found to result in premature thymic aging (102).

While the mechanisms via which PiT1 impacts on cell cycle and hematopoietic lineage differentiation have still not been fully elucidated, it appears that the ERK1/2 pathway is involved in this process. Specifically, increases in extracellular phosphate have been shown to induce ERK1/2 signaling and upregulation of cyclin D1 (103, 104). While ERK1/2 phosphorylation has been linked to phosphate uptake, more recent data suggest that activation of this pathway is mediated by a phosphate-regulated heterodimerization of PiT1 and PiT2, independently of phosphate uptake. Indeed, deficient ERK1/2 phosphorylation in PiT-1 or PiT2-depleted cells was rescued by transport-deficient PiT mutants (53). Thus, the impact of phosphate sensing by PiT1 and PiT2, can potentially modulate thymocyte differentiation in a manner that is independent of phosphate uptake. Moreover, as robust ERK activation has been shown to be associated with thymocyte death whereas a low/brief ERK activation is associated with positive selection (105–107), it will be of much interest to study the impact of PiT2 on ERK activation in thymocytes and determine its potential role in positive selection.

Our finding that PiT1 expression profiles are altered in the aging thymus adds to our understanding of thymic involution and alterations in thymocyte subsets as a function of age. The percentage of CD3^+^DN cells expressing the PiT1 transporter increased by 5-fold between 2 weeks and 1 year of age. One subset of CD3^+^DN thymocytes represents DP thymocytes with an autoreactive TCR wherein strong TCR signaling directs them to a CD3^+^CD44^+^PD1^+^DN stage of differentiation (89). However, consistent with a decreased differentiation and metabolism in older mice, the percentages of these CD3^+^DN thymocytes expressing PD1 or the CD71 transferrin receptor was significantly diminished and they did not represent the PiT1^+^ subset. Interestingly though, ~50% of PD1^−^CD44^+^CD25^−^CD3^+^DN thymocytes, a subset that was previously shown to accumulate in the thymus of older mice (85, 108–111), exhibited high levels of PiT1 by 1 year of age. CD44^+^CD3^+^DN thymocytes can represent γδ or NKT thymocytes (90–94) and as such, we evaluated both these subsets. While γδ thymocytes did not increase with age, NKT cells were significantly augmented. This NKT subset was specific to the CD3^+^DN population as expression of NK1.1 on CD3^Int^CD4SP thymocytes was low (6 ± 5%), did not change with age, and did not exhibit high PiT1 expression (Supplementary Figure 7B). The significance of high PiT1 levels in this CD3^+^DN thymocyte subset is at present unknown but it will be of much interest to determine whether PiT1 expression on γδ or NKT thymocytes alters their function and/or can serve as a biomarker of thymic aging. Furthermore, the profile of PiT1 expression in responses to thymic insults such as irradiation and chemotherapy, resulting in alterations in the extracellular thymus niche, is not known. Indeed, the impact of toxic insults, and conversely, thymic regeneration strategies such as IL-22 or chemical castration (84, 112, 113), may alter the dynamic expression profiles of PiT1 as well as PiT2.

Differences in the profiles of the PiT1 and PiT2 transporters in the murine and human thymus suggest that they play distinct roles in thymocyte differentiation and proliferation. Conditional loss of PiT1, albeit with suboptimal deletion of the floxed allele, did not inhibit murine thymocyte differentiation (65). These data are consistent with the low-level expression of PiT1 that we detected in the thymi of young mice, and especially in immature thymocytes. In contrast with homozygous PiT1 deletion which results in embryonic lethality (55), mice with deleted PiT2 are viable and develop brain calcifications (56). The pathophysiology of these mice resembles the pathology of patients with primary familial brain calcification (PFBC). Indeed, 40% of patients with this neurodegenerative disease harbor mutations in PiT2 (58). While thymocyte differentiation has not been evaluated in these mice, the high level of PiT2 in immature thymocytes progressing through the β-selection checkpoint suggests that thymocyte differentiation would be negatively affected, as detected in mice with a conditional deletion of GLUT1 (37). In support of a potential role for PiT2 in early thymus differentiation, deletion of genes that alter thymocyte metabolism, such as apoptosis-inducing factor (AIF) (114) and SdhD (115), have resulted in a block in thymocyte differentiation, at the DN3/DN4 transition. Finally, it will be of interest to determine whether patients with PFBC exhibit decreased thymocyte differentiation, evaluated as a function of TRECs, especially given the association in several genetic immunodeficiencies with CNS involvement (116).

Our data highlight distinct profiles of PiT1 and PiT2 in murine and human thymus, revealing developmental specificities in the expression of these phosphate transporters. Furthermore, increased PiT1 levels on CD3^+^DN thymocytes, identified in majority as NKT thymocytes, was found to be a biomarker of thymi of >1 year of age. Our study also identifies PiT2/SLC20A2 as a member of the family of metabolite transporters that characterizes immature thymocytes with high metabolic activity and the upregulation of PiT2 at the beta-selection checkpoint is conserved between mouse and man. The list of metabolite transporters that are induced at the beta-selection checkpoint, now comprising PiT2, GLUT1 and CD71, is likely to grow—pointing to an extensive metabolic crosstalk regulating the proliferation and differentiation of immature thymocytes.

## Data Availability Statement

The raw data supporting the conclusions of this article will be made available by the authors, without undue reservation.

## Ethics Statement

The animal studies were reviewed and approved by the Languedoc-Rousillon Animal Care Committee, Montpellier, France and the NCI Animal Care Committee, Bethesda, MD.

## Author Contributions

VZ and NT conceived and supervised the study. AM, MP, SG, VF, MC, VZ, and NT were involved in study design. AM, MP, SG, VF, MC, UL-S, VP, and VZ performed experiments. TK, FP, MB, and LN contributed to sample preparation and quality control. AM, MP, VZ, and NT wrote the manuscript and all authors critically reviewed the manuscript. All authors participated in data analysis and discussions.

## Conflict of Interest

VP and NT are inventors on patents describing the use of RBD ligands but NT no longer has any patent rights. VP is the co-founder of METAFORA-biosystems, a start-up company that focuses on metabolite transporters under physiological and pathological conditions. The remaining authors declare that the research was conducted in the absence of any commercial or financial relationships that could be construed as a potential conflict of interest.

## Supplementary Material

Supplementary Figure 1 Gating strategies for evaluation of murine and human thymocyte subsets. **(A)** Gating strategy for murine thymocytes showing FSC/SSC profiles, followed by SSC-W/SSC-H, FSC-W/FSC-H and Live-Dead analysis. Non-T lineage thymocyte subsets were eliminated by a ≪ Dump ≫ staining with anti- CD11b, -CD19, -Ter119, and -Gr1 mAbs. CD4/CD8 profiles as well as profiling of DN thymocytes and CD8^+^ thymocytes (CD3-ISP and CD3^+^ SP8) are shown. **(B)** Profiles for live freshly isolated CD4-selected T cells. For evaluation of naïve and memory CD4 T cells, CD45RA/CD45RO profiles are presented. **(B)** Gating strategy for human thymocytes showing FSC/SSC profiles, followed by SSC-W/SSC-H, FSC-W/FSC-H and Live-Dead analysis. Representative CD4/CD8 profiles and evaluation of CD4^+^ thymocytes (CD3-ISP and CD3^+^SP4) are shown. The percentages of cells in each gate are indicated.

Supplementary Figure 2 Specificity of retroviral envelope binding domain binding to PiT1 and PiT2 phosphate transporters. **(A)** BxPC3 cells were transduced with a PiT1 specific shRNA and PiT1 expression in parental and shRNA-transduced cells was monitored with the Koala retroviral envelope receptor binding domain (Ko-RBD, Metafora Biosystems). A representative histogram with non-specific binding in gray, parental in blue and shRNA knockdown in red is shown (left) and quantification from three independent experiments was evaluated by molecules of equivalent soluble fluorochrome (MESF). **(B)** The specificity of the amphotropic MLV RBD for evaluation of PiT2 expression was evaluated as previously reported (69), monitoring binding of the ampho-delta SU construct (A-RBD) in the parental haploid HAP1 cell line as well as following CRISPR-mediated knockout of PiT2 (HAP1- DPiT2). Representative histograms showing non-specific (line) and specific (gray) staining are presented. Note that there is an auto-fluorescence in HAP1- DPiT2 cells relative to the non-specific staining.

Supplementary Figure 3 GLUT1 and PiT1 expression profiles on Foxp3^+^ thymocytes. Expression of GLUT1 and PiT2 was evaluated in CD25^−^Foxp3^+^ and CD25^+^Foxp3^+^ thymocytes (left) and representative histograms are shown (middle). The percentages of cells in each gate are indicated. Quantification of GLUT1 and PiT2 detection in the CD25^−^Foxp3^+^ and CD25^+^Foxp3^+^ subsets are presented (*n* = 5, left).

Supplementary Figure 4 Profiles of TN thymocytes in WT, Pmel-1 and RAG2^−/−^ mice. Expression profiles of TN thymocytes from WT, Pmel-1 and RAG2^−/−^ mice, distinguishing TN1, TN2, TN3, and TN4 thymocytes as a function of CD44^+^CD25^−^, CD44^+^CD25^+^, CD44^−^CD25^+^, and CD44^−^CD25^−^ staining, are presented.

Supplementary Figure 5 Detection of GLUT1, PiT1, and PiT2 on human thymocytes. The detection of GLUT1, PiT1, and PiT2 on human thymocytes was evaluated and representative histograms, indicating the percentages of positively stained cells, are shown (left). Quantification of percentages with horizontal lines presenting means ±SD are presented (*n* = 4 from three independent thymi, representative of five independent thymus specimens; right).

Supplementary Figure 6 Lack of CD71 transferrin receptor expression on PiT1^+^CD3^+^DN thymocytes. Expression of PiT1 and CD71 was evaluated on CD3^+^DN thymocytes from 2 weeks, 8 weeks, and 1yo mice. Representative dot plots are presented (left) and quantification of CD71 expression in the different age groups is shown (right).

Supplementary Figure 7 PiT1 but not GLUT1 or PiT2 is expressed on NK1.1^+^ thymocytes. **(A)** Expression of GLUT1, PiT1, and PiT2 were evaluated within NK1.1^+^ thymocytes in the DN gate and representative histograms as well as percent positively staining cells are shown (left). Quantification of GLUT1, PiT1, and PiT2 in NK1.1^+^ thymocytes is shown with horizontal lines presenting means ±SD (*n* = 5, right). **(B)** CD4^+^ thymocytes were evaluated as a function of intermediate and high CD3 expression at 8 weeks and 1 year of age (left plots), and NK1.1/PiT1 staining profiles were evaluated in both subsets (middle plots). Quantification of NK1.1 staining within the CD3^int^CD4^+^ thymocyte subset is shown (*n* = 5, right).
