# Supplementary figures and images for: Phosphate Transporter Profiles in Murine and Human Thymi Identify Thymocytes at Distinct Stages of Differentiation
Source: Front Immunol. 2020 Jul 22;11:1562. doi: 10.3389/fimmu.2020.01562 (PMC7387685; doi:10.3389/fimmu.2020.01562)

Supplemental FIGURE 1

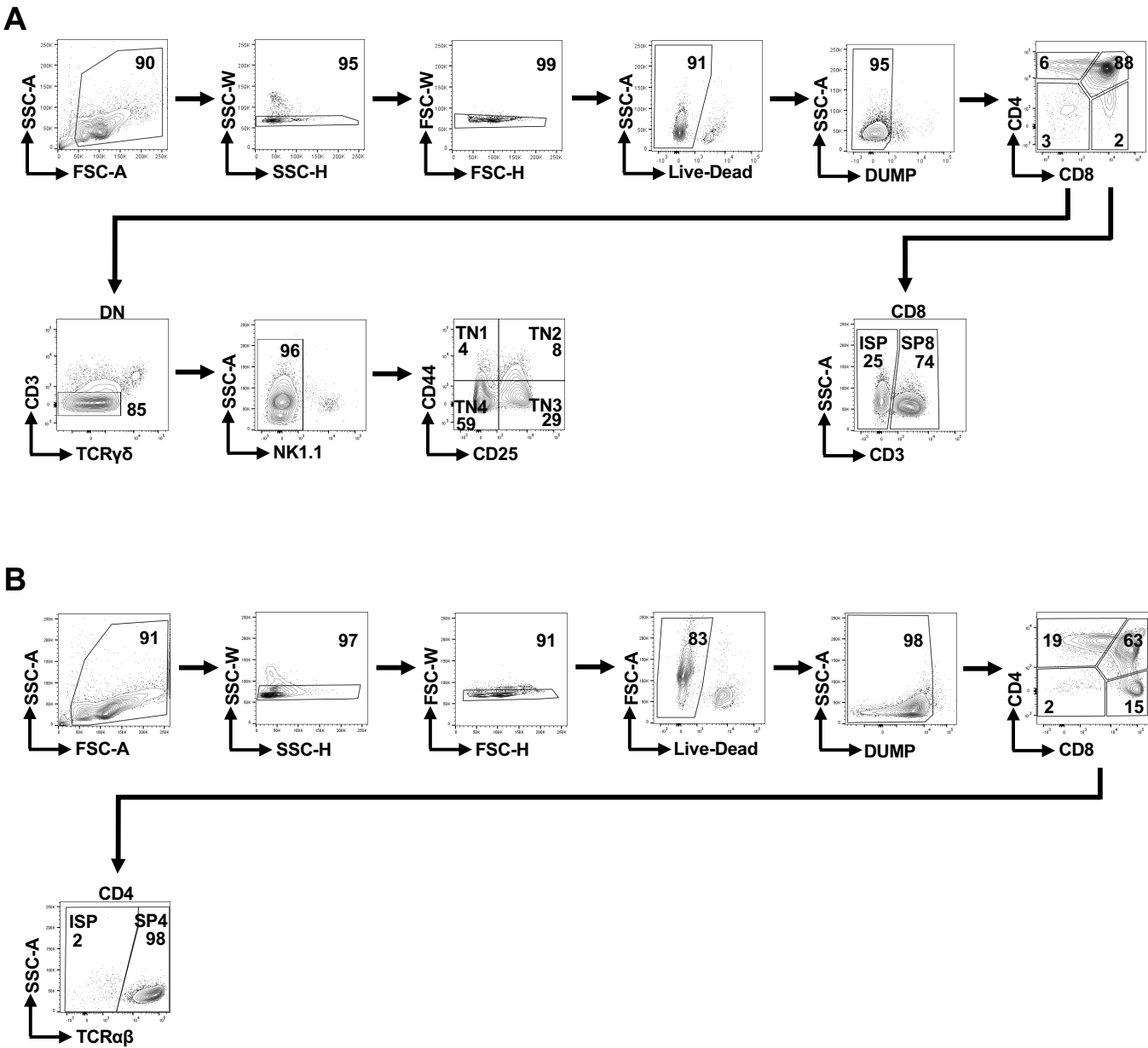

Supplement: Supplementary Figure 1 — Gating strategies for evaluation of murine and human thymocyte subsets. (A) Gating strategy for murine thymocytes showing FSC/SSC profiles, followed by SSC-W/SSC-H, FSC-W/FSC-H and Live-Dead analysis. Non-T lineage thymocyte subsets were eliminated by a ≪ Dump ≫ staining with anti- CD11b, -CD19, -Ter119, and -Gr1 mAbs. CD4/CD8 profiles as well as profiling of DN thymocytes and CD8+ thymocytes (CD3-ISP and CD3+ SP8) are shown. (B) Profiles for live freshly isolated CD4-selected T cells. For evaluation of naïve and memory CD4 T cells, CD45RA/CD45RO profiles are presented. (B) Gating strategy for human thymocytes showing FSC/SSC profiles, followed by SSC-W/SSC-H, FSC-W/FSC-H and Live-Dead analysis. Representative CD4/CD8 profiles and evaluation of CD4+ thymocytes (CD3-ISP and CD3+SP4) are shown. The percentages of cells in each gate are indicated. [file Image_1.pdf]

Supplemental FIGURE 2

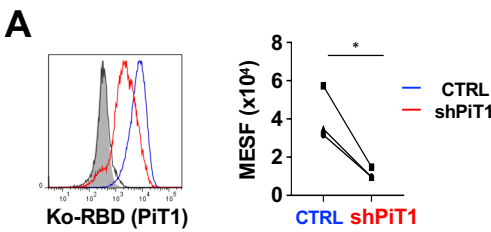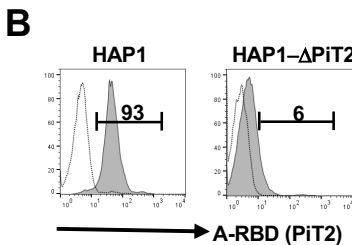

Supplement: Supplementary Figure 2 — Specificity of retroviral envelope binding domain binding to PiT1 and PiT2 phosphate transporters. (A) BxPC3 cells were transduced with a PiT1 specific shRNA and PiT1 expression in parental and shRNA-transduced cells was monitored with the Koala retroviral envelope receptor binding domain (Ko-RBD, Metafora Biosystems). A representative histogram with non-specific binding in gray, parental in blue and shRNA knockdown in red is shown (left) and quantification from three independent experiments was evaluated by molecules of equivalent soluble fluorochrome (MESF). (B) The specificity of the amphotropic MLV RBD for evaluation of PiT2 expression was evaluated as previously reported (69), monitoring binding of the ampho-delta SU construct (A-RBD) in the parental haploid HAP1 cell line as well as following CRISPR-mediated knockout of PiT2 (HAP1- DPiT2). Representative histograms showing non-specific (line) and specific (gray) staining are presented. Note that there is an auto-fluorescence in HAP1- DPiT2 cells relative to the non-specific staining. [file Image_2.pdf]

Supplemental Figure 3

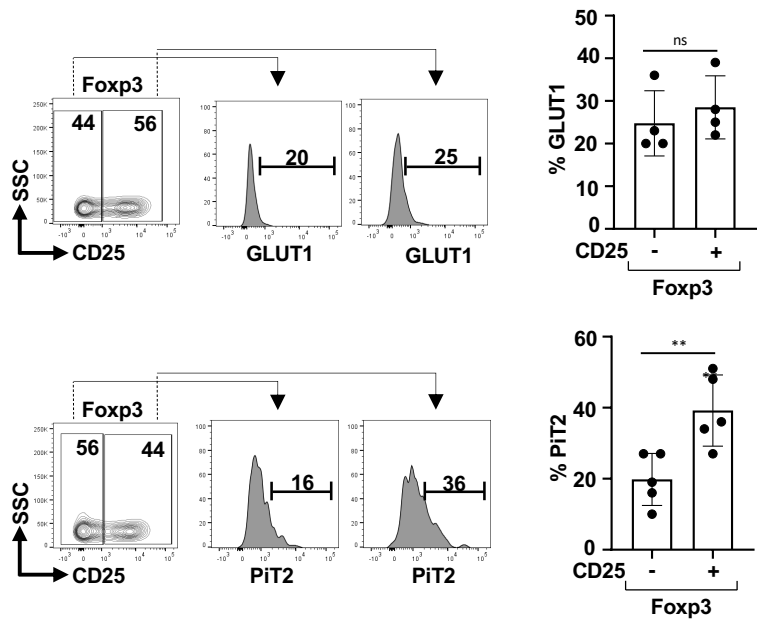

Supplement: Supplementary Figure 3 — GLUT1 and PiT1 expression profiles on Foxp3+ thymocytes. Expression of GLUT1 and PiT2 was evaluated in CD25−Foxp3+ and CD25+Foxp3+ thymocytes (left) and representative histograms are shown (middle). The percentages of cells in each gate are indicated. Quantification of GLUT1 and PiT2 detection in the CD25−Foxp3+ and CD25+Foxp3+ subsets are presented (n = 5, left). [file Image_3.pdf]

Supplemental Figure 4

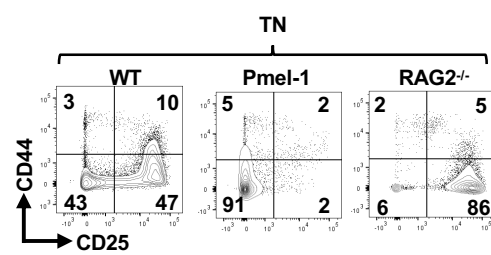

Supplement: Supplementary Figure 4 — Profiles of TN thymocytes in WT, Pmel-1 and RAG2−/− mice. Expression profiles of TN thymocytes from WT, Pmel-1 and RAG2−/− mice, distinguishing TN1, TN2, TN3, and TN4 thymocytes as a function of CD44+CD25−, CD44+CD25+, CD44−CD25+, and CD44−CD25− staining, are presented. [file Image_4.pdf]

Supplemental Figure 5

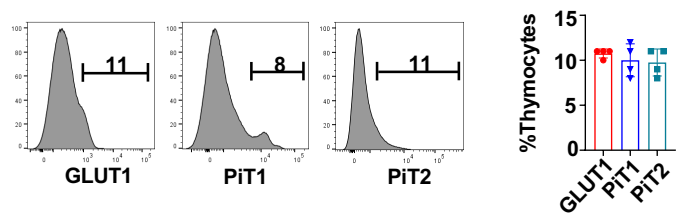

Supplement: Supplementary Figure 5 — Detection of GLUT1, PiT1, and PiT2 on human thymocytes. The detection of GLUT1, PiT1, and PiT2 on human thymocytes was evaluated and representative histograms, indicating the percentages of positively stained cells, are shown (left). Quantification of percentages with horizontal lines presenting means ±SD are presented (n = 4 from three independent thymi, representative of five independent thymus specimens; right). [file Image_5.pdf]

Supplemental Figure 6

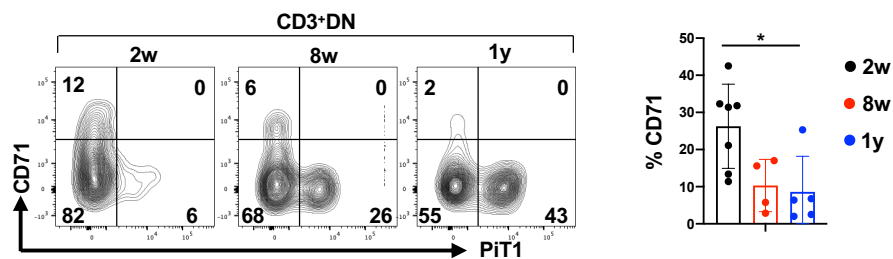

Supplement: Supplementary Figure 6 — Lack of CD71 transferrin receptor expression on PiT1+CD3+DN thymocytes. Expression of PiT1 and CD71 was evaluated on CD3+DN thymocytes from 2 weeks, 8 weeks, and 1yo mice. Representative dot plots are presented (left) and quantification of CD71 expression in the different age groups is shown (right). [file Image_6.pdf]

Supplemental FIGURE 7

A

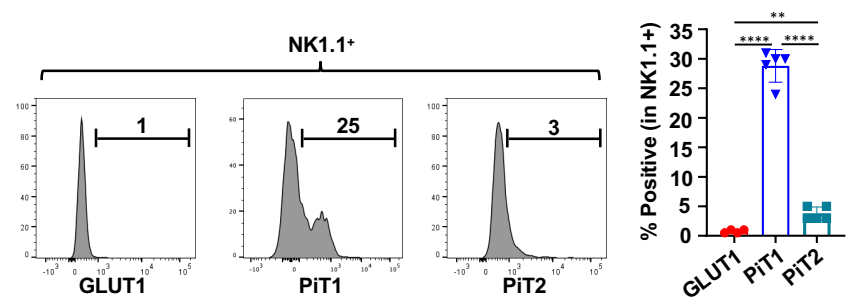

B

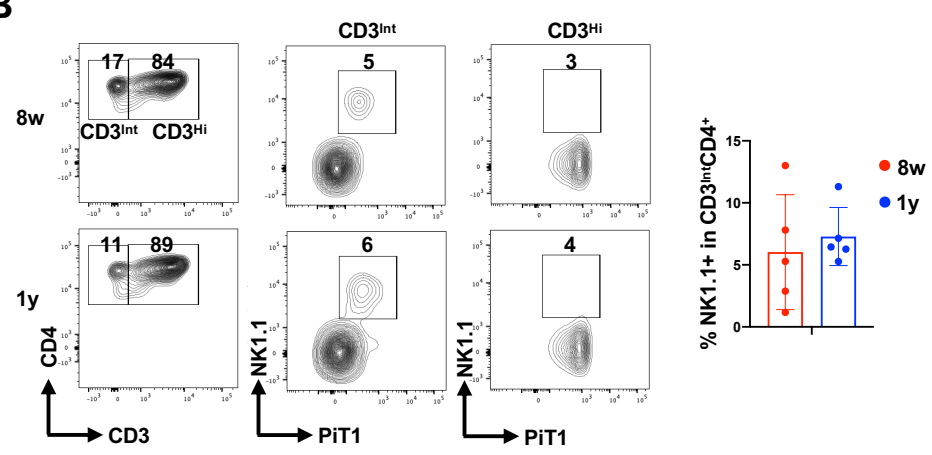

Supplement: Supplementary Figure 7 — PiT1 but not GLUT1 or PiT2 is expressed on NK1.1+ thymocytes. (A) Expression of GLUT1, PiT1, and PiT2 were evaluated within NK1.1+ thymocytes in the DN gate and representative histograms as well as percent positively staining cells are shown (left). Quantification of GLUT1, PiT1, and PiT2 in NK1.1+ thymocytes is shown with horizontal lines presenting means ±SD (n = 5, right). (B) CD4+ thymocytes were evaluated as a function of intermediate and high CD3 expression at 8 weeks and 1 year of age (left plots), and NK1.1/PiT1 staining profiles were evaluated in both subsets (middle plots). Quantification of NK1.1 staining within the CD3intCD4+ thymocyte subset is shown (n = 5, right). [file Image_7.pdf]
